# Retraction Note: BCORL1 is an independent prognostic marker and contributes to cell migration and invasion in human hepatocellular carcinoma

**DOI:** 10.1186/s12885-023-11051-6

**Published:** 2023-06-13

**Authors:** Guozhi Yin, Zhikui Liu, Yufeng Wang, Changwei Dou, Chao Li, Wei Yang, Yingmin Yao, Qingguang Liu, Kangsheng Tu

**Affiliations:** Department of Hepatobiliary Surgery, the First Affiliated Hospital of Xi’an Jiaotong University, No.277 Yanta West Road, Xi’an, 710061 China

**Retraction Note: BMC Cancer 16, 103 (2016)**

10.1186/s12885-016-2154-z

The Editors have retracted this article because of irregularities in Figure 3, specifically:

- In Figure 3B the panel Migration vs. Scrambled siRNA overlaps with the panel Invasion vs. Scrambled siRNA.
- In Figure 3D the panel Migration vs. EV overlaps with the panels Invasion vs. EV and Invasion vs. BCORL1.

The Editors therefore no longer have confidence in the veracity of the data presented. None of the authors has responded to correspondence about this retraction.

